# Advancements in plant based meat analogs enhancing sensory and nutritional attributes

**DOI:** 10.1038/s41538-024-00292-9

**Published:** 2024-08-07

**Authors:** Jiwon Jang, Dong-Woo Lee

**Affiliations:** 1 Graduate Program in Bio-industrial Engineering, Yonsei University, Seoul, 03722 South Korea; 2 Department of Biotechnology, Yonsei University, Seoul, 03722 South Korea

**Keywords:** Biomaterials - proteins, Biomaterials - proteins

## Abstract

The burgeoning demand for plant-based meat analogs (PBMAs) stems from environmental, health, and ethical concerns, yet replicating the sensory attributes of animal meat remains challenging. This comprehensive review explores recent innovations in PBMA ingredients and methodologies, emphasizing advancements in texture, flavor, and nutritional profiles. It chronicles the transition from soy-based first-generation products to more diversified second- and third-generation PBMAs, showcasing the utilization of various plant proteins and advanced processing techniques to enrich sensory experiences. The review underscores the crucial role of proteins, polysaccharides, and fats in mimicking meat’s texture and flavor and emphasizes research on new plant-based sources to improve product quality. Addressing challenges like production costs, taste, texture, and nutritional adequacy is vital for enhancing consumer acceptance and fostering a more sustainable food system.

## Introduction

**Fig. 1:**
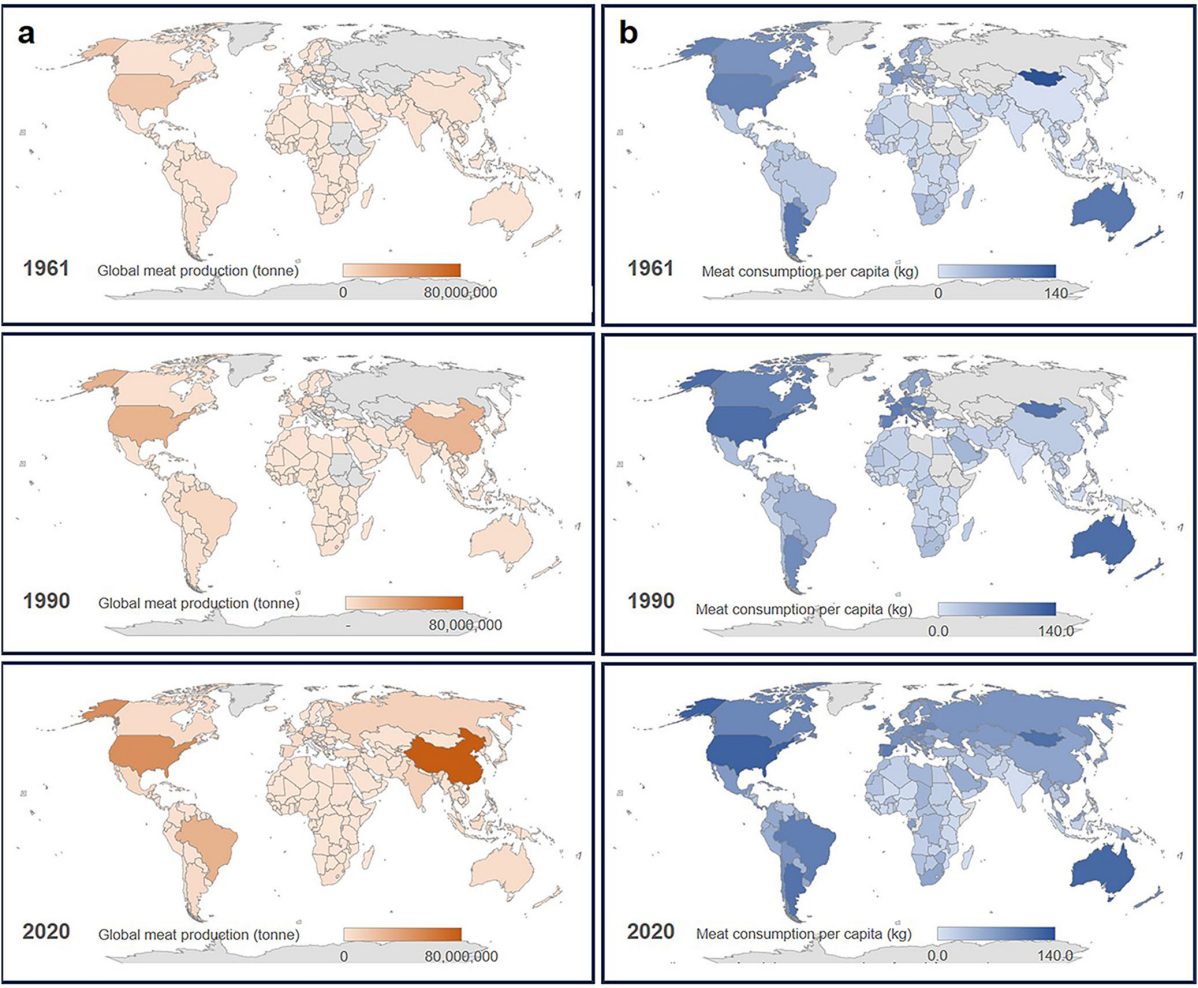
Global trends in meat production and consumption (1960–2020). **a** Global mean meat production from 1960 to 2020. In 1961, 12 (7.3%) of 164 countries produced more than a million tonnes of meat, whereas by 2020, 46 (10.9%) of 193 countries surpassed this production milestone. **b** The global average meat consumption per capita from 1960 to 2020. In 1961, 5 (2.0%) of 250 countries had a meat consumption of at least 100 kg/year per capita, accounting for 0.037 billion people. By 1990, 9 (3.42%) of 263 countries reached this consumption level, corresponding to 0.32 billion people. In 2020, 9 (3.57%) out of 252 countries reported per capita intakes of at least 100 kg, representing 1.06 billion people. Source: Our World in Data (https://ourworldindata.org).

Over recent decades, the global meat industry has experienced substantial growth. By 2020, meat production reached around 337 million tons, nearly five times the amount produced in the 1960s^1^. Europe and North America initially led production, but by 2020, Asia became the major contributor, accounting for 41% of global meat production (Fig. 1a). This growth is attributed to the doubling of the global population and significant socio-economic changes. Projections suggest that by 2050, as the global population approaches 9 billion, demand for meat production could increase by 50–73%^2^. This trend is due to population growth and a tripling in global income over the past fifty years, making meat more accessible and increasing its consumption^3^ (Fig. 1b). The livestock sector, including meat and dairy, significantly impacts the environment, contributing to deforestation, greenhouse gas (GHG) emissions, and water pollution^4^. Additionally, high red meat consumption is linked to health risks like cardiovascular diseases, colorectal cancers, and type 2 diabetes^5^. Conventional meat production also raises animal welfare concerns, often involving inhumane practices^6^.

The meat consumption and production challenges are multifaceted, encompassing food security, health risks, and animal welfare concerns^7^. About 800 million people globally face chronic hunger, with another 2 billion suffering from micronutrient deficiencies^8^. The COVID-19 pandemic highlighted vulnerabilities in the food supply chain, underscoring the need for resilient and sustainable food systems^9^. While meat is a vital nutrient source, excessive consumption, mainly processed meats, is associated with various health complications^10^. Acknowledging these unsustainable practices, the Food and Agriculture Organization (FAO) advocates a shift towards plant-based diets to mitigate climate change impacts and promote sustainable food ecosystems^11^. Innovative alternatives like plant-based substitutes and cultured meats have emerged, offering more sustainable and ethical food choices. Plant-based meat analogs (PBMAs) have gained attention for their potential to provide a more nutritious profile than red meat. These alternatives aim to replicate the texture and flavor of animal meats using plant-derived ingredients, but they face challenges in authentically replicating conventional meat^12^. This review seeks to navigate these challenges, examining the latest trends, ingredients, and innovative methods in the meat alternative sector.

## Types of alternative meats

**Fig. 2:**
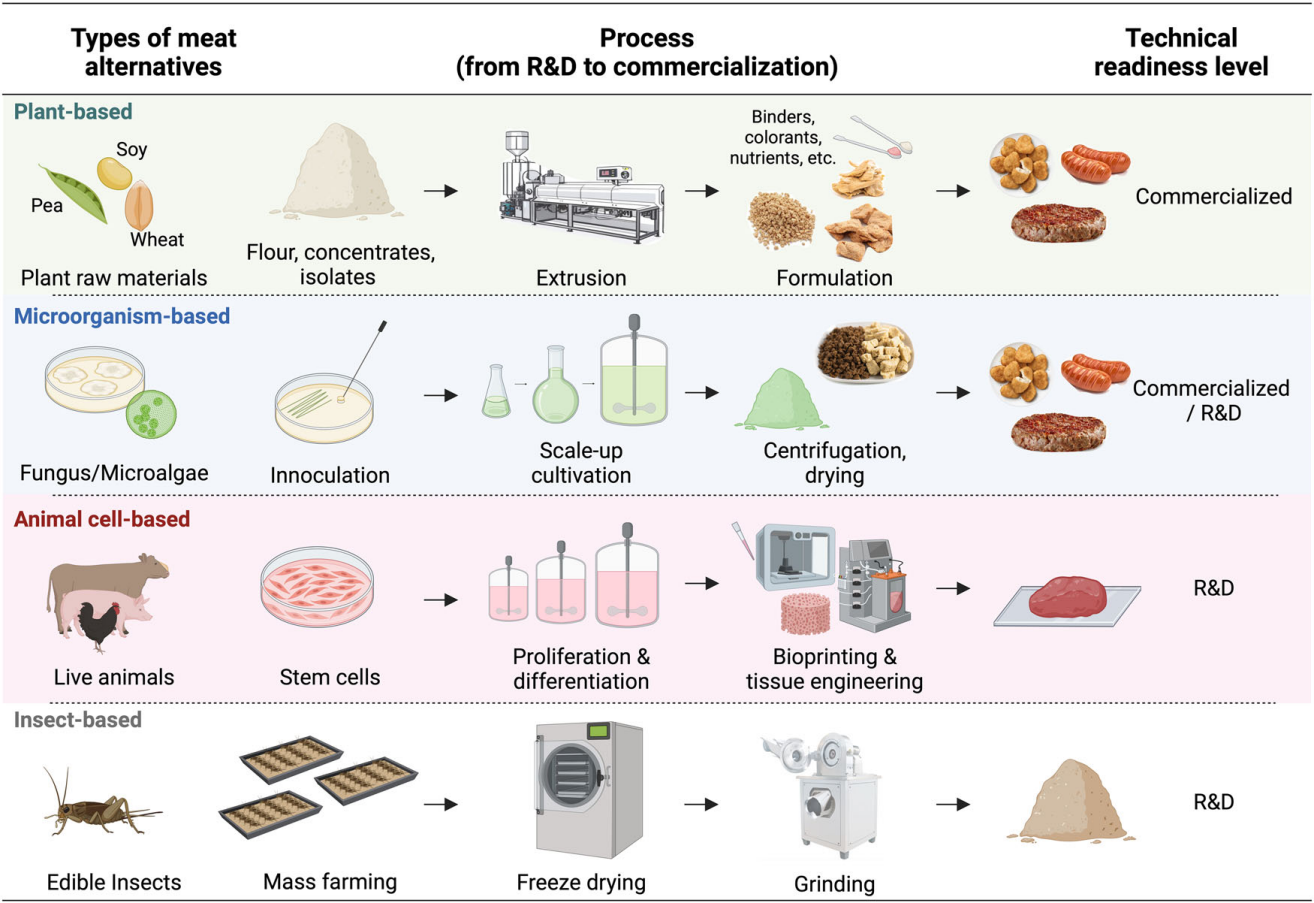
Meat alternatives: types and advancements in production technology. This infographic delineates the production process of different types of meat alternatives.

Alternative meats encompass four primary categories based on their origin: plant-based, microorganism-based, animal-based, and insect-based meat analogs, each with distinctive sources and production processes (Fig. 2).

### Plant-based meat analogs (PBMAs)

PBMAs, crafted mainly from legumes, soybeans, wheat, and lentils, mimic the fibrous texture of meat using protein reforming techniques like extrusion, shear cells, and three-dimensional (3D) printing^13^. These analogs, considered healthier and more eco-friendly due to lower calorie and saturated fat content, still face challenges in preserving nutritional value and cost-effectiveness during intensive processing, which may lead to increased salt content and reduced essential micronutrients^14^. Nevertheless, PBMAs are gaining commercial momentum, with ongoing production methods and raw material selection improvements to enhance their sensory and nutritional appeal.

### Microorganism-based meat analogs (MBMAs)

MBMAs, particularly mycoprotein from fungi like *Fusarium venenatum*, offer rich protein and fiber content and notable health benefits^15^. Production involves using agricultural and industrial by-products and crucial freezing stages for meat-like texture^16^. Despite challenges like resource-intensive production and allergenicity, microalgae like Chlorella and Spirulina are gaining attraction in PBMA applications due to their rich nutrient profiles and functional properties^17^. However, they face hurdles related to color, odor, and texture in PBMA integration^18^.

### Animal cell-based meat analogs (ABMAs)

Lab-grown meat, an eco-friendly alternative, is produced by cultivating stem cells from muscle tissue or embryos in bioreactors^19^, structured on edible scaffolds^20^, and formed into muscle fibers using tissue engineering techniques^21^. Since the first lab-grown hamburger debuted in 2013, significant advancements have been made in scaffold technologies, including 3D bioprinting^22^ and electrospinning^23^. Despite its potential to mimic meat’s sensory attributes and recent regulatory milestones^24^, cultured meat still faces challenges in fat composition modification^25^, nutrient content adjustment^26^, technical complexities like co-culturing different cell types^27^, scalability, production costs, and public acceptance^28^.

### Insect-based meat analogs (IBMAs)

Insect proteins from crickets, mealworms, ants, and black soldier flies offer a promising alternative rich in nutrients like amino acids and bioactive peptides^29^. Despite their potential and global inclusion in diets, concerns about safety, shelf-life, and consumer acceptance necessitate stringent standards, thorough quality checks, and proper regulation. Addressing health risks, ensuring safe preparation^30^, and devising strategies to increase consumer acceptance, such as incorporating insects into familiar foods or inconspicuous powdered forms, are pivotal for advancing IBMAs^31^.

## Classification and evolution of PBMAs

**Fig. 3:**
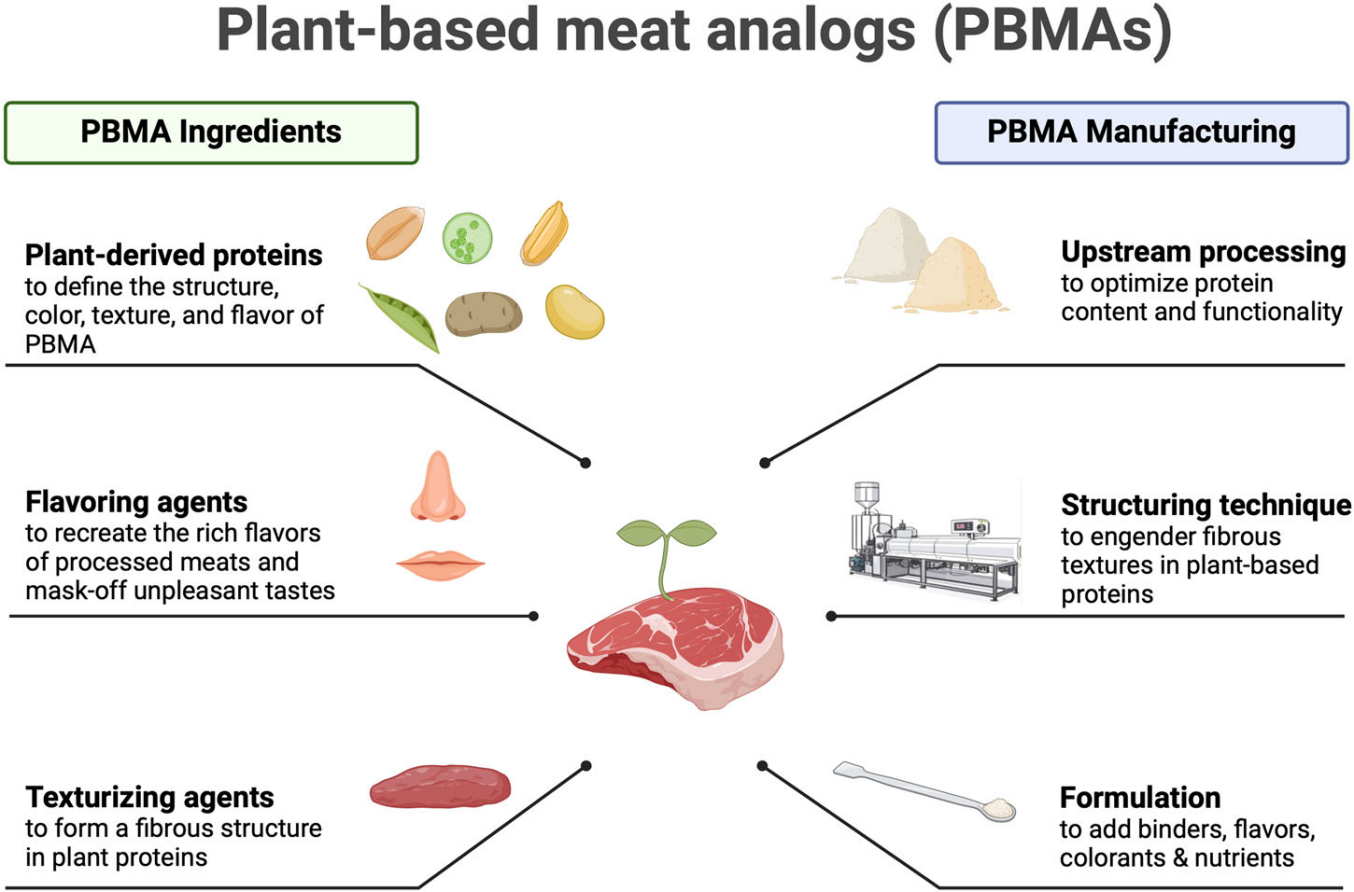
Overview of PBMAs production process. This infographic provides a detailed view of the PBMA production steps. The left side details the key ingredients: plant-derived proteins that shape texture and flavor, flavoring agents that enhancing taste while masking off-flavors, and texturizing agents for meat-like fibrous structures. The right-side outlines manufacturing phases, from upstream protein optimization and various structuring techniques to create meat-like textures, to final formulation steps integrating binders, flavors, colorants, and nutrients.

The development of PBMAs has seen significant advancements over time, evolving through several generations of products and innovations (Fig. 3). Initially dominated by soy protein, the sector expanded to diverse plant-based proteins, leveraging ingredients such as pea, wheat, potato, mung bean, and rice (Table 1). This journey from traditional products to advanced alternatives highlights the plant-based meat industry’s commitment to addressing consumer preferences and environmental concerns, continually evolving to overcome earlier limitations.

**Table 1:** Classification and characterization of PBMAs

Class	Source		Key features	Generation	Global Production Amount (MMT)^a^	References
**Common protein ingredients**	Soy		• Good gelation effect for extrusion • Strong emulsifying properties	1^st^	>100^b^	^136^
	Pea		• High-quality protein with 23–35% • Neutral flavor profile	1^st^	1–9^b^	^137^
	Wheat		• Viscoelastic property to form a network • Hardness and chewiness improved in PBMAs	1^st^	>100^b^	^56^
	Potato		• Emulsifying property contributing to final products’ mouthfeel • Lowest allergy rate	2^nd^	>100^c^	^138^
	Rice		• Hypoallergenic and high biological value • Well-balanced amino acid composition	2^nd^	>100^b^	^58^
**Novel proteinaceous ingredients**	Pulse	Lentil	• Gelling, emulsifying, and foaming properties	3^rd^		^60^
		Mungbean	• Excellent gelling properties and high water-holding capacity	3^rd^	1–9^b^	^60^
		Faba bean	• Higher biological nitrogen fixation potential	3^rd^	1–9^b^	^61,139^
	Oilseeds	Pumpkin seeds, flaxseeds, chia seeds, etc.	• Low content of anti-nutritional factors • Antioxidant activity, anti-inflammatory, and antibacterial effect	3^rd^	10–99^d^	^63,140^
	Pseudocereal	Amaranth	• Gelling properties • Balanced amino acid profile and good bioavailability	3^rd^	–	^64,141^
		Quinoa	• Emulsifying and gelling properties • Balanced amino acid profile	3^rd^	<1^e^	^65,142^
	Algae	Seaweed	• High protein, dietary fiber, vitamins and minerals	3^rd^	10–99^f^	^66^
	Aquatic plant	RuBisco (Duckweed)	• Foaming, gelling, and emulsifying properties • Easily digestible and nutritionally complete	3^rd^		^68^

^a^MMT stands for a million metric ton and is 1,000,000,000 kg.

^b^https://gfi.org/.

^c^https://www.potatonewstoday.com/.

^d^https://www.statista.com/.

^e^https://www.statista.com/.

^f^https://openknowledge.fao.org/.

### The 1st generation of plant-based meat

Initially, the PBMA sector relied on traditional Asian protein sources like tofu and tempeh, known for their unique textures and rich nutrient profiles^32^. Tempeh from Indonesia, for example, effectively emulates meat texture, while yuba, a Japanese culinary product, serves as an eco-friendly wrapping material^33^. Innovations such as textured vegetable protein (TVP), seitan, and mycoprotein emerged to meet the rising demand from vegetarians and those preferring plant-based diets. TVP, processed via low moisture extrusion (LME), is designed to achieve a meat-like texture when rehydrated^34^. Seitan, made from vital wheat gluten, offers a chewy texture similar to meat. Mycoprotein, marketed as Quorn by Marlow Foods in 1985, quickly gained popularity as a meat substitute^35^. These early products, foundational to today’s robust plant-based meat industry, often lacked the complex texture and flavor diversity of animal meat, offering a somewhat limited sensory experience^34^. For example, replacing 30% (w/w) of beef with TVP in sausages adjusted the water content, brightness, and yellowness, decreased fat content and hardness, thereby affecting sensory attributes^36^. Additionally, the texture of PBMA patties, characterized by lesser hardness, chewiness, and gumminess compared to real meat, was to some extent improved by incorporating additives like methylcellulose to boost taste and flavor^37^.

### The 2nd generation of plant-based meat

**Fig. 4:**
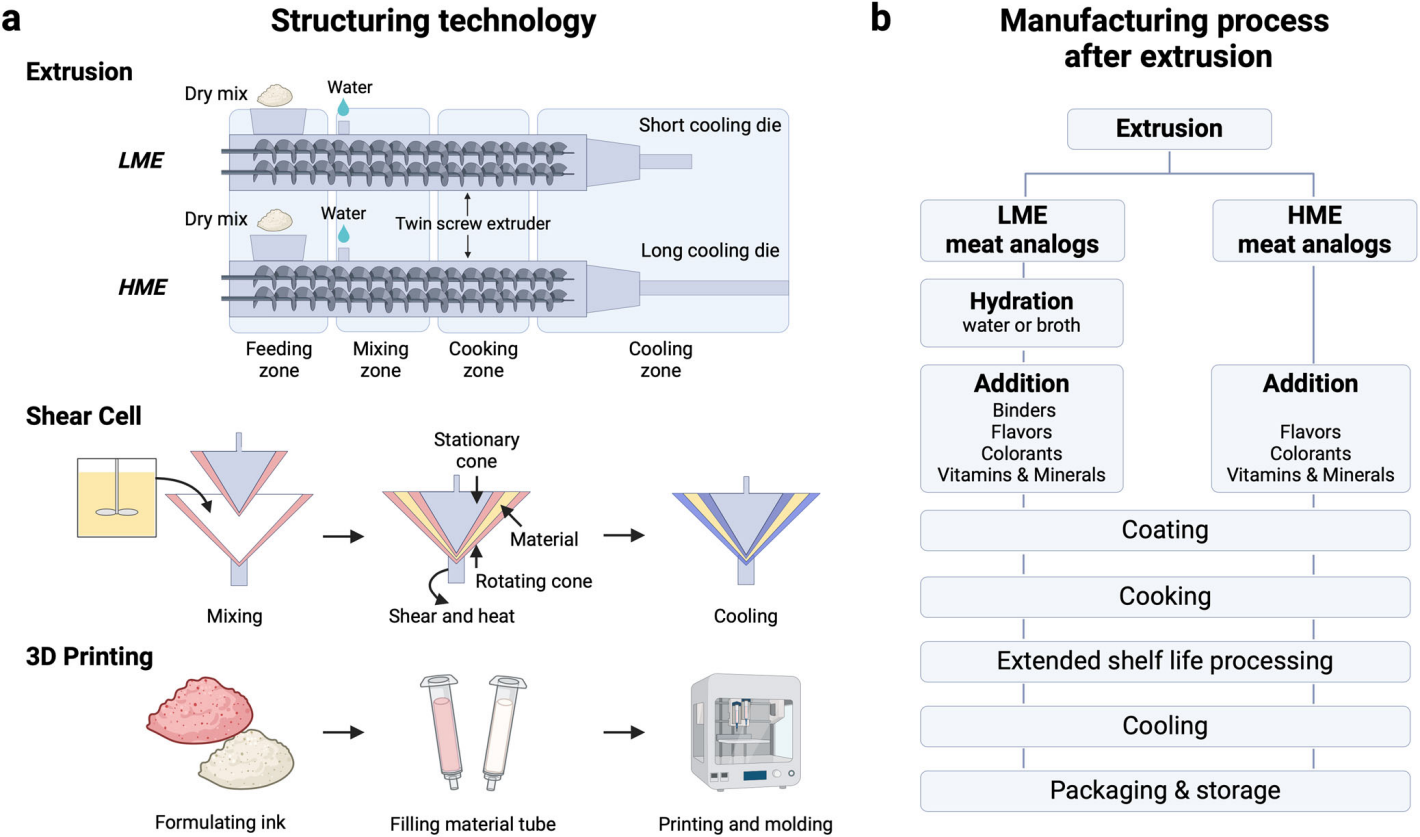
Innovations in PBMA production. **a** Structuring technologies are employed to emulate meat-like texture and shape. **b** Schematic representation of the manufacturing process for PBMAs using extrusion.

The second generation of plant-based meats aimed to more closely replicate the fibrous texture and flavor of animal-based meats, leveraging advanced technologies such as high-moisture extrusion (HME), shear cells, and 3D printing^38^. These approaches range from bottom-up methods such as microbial fermentation^39^, electrospinning^23^, and cultured meat^22^, which integrate aligned fibers with various ingredients, to top-down methods including extrusion^40^, freezing^41^, and shear technology that create fibrous textures by stretching biopolymer blends^42^. Bottom-up methods often face challenges in accurately replicating muscle structure and achieving scalability. Conversely, HME has proven highly effective, employing intense mechanical mixing under high pressures and temperatures to transform plant-based protein isolates into products that closely mimic the texture and flavor of meat^43^. The HME process involves several stages within the extruder (Fig. 4a): starting with a low-temperature mixing zone that hydrates proteins without altering their structure, moving to a moderate-temperature zone for partial denaturation, and culminating in a high-temperature melting zone where proteins fully denature^44^. Controlled cooling solidifies the melt, facilitating phase separation and bond formation to finalize the fibrous structure^45^. Leading brands like Marlow Foods, Gardein, Beyond Meat, and Impossible Foods have continuously refined their offerings, significantly narrowing the gap between plant-based and traditional meat products.

### The 3rd generation of plant-based meat

The third generation emerged in response to environmental concerns and a surge in veganism, particularly during the COVID-19 pandemic. This generation debunks myths about plant-based meats being inherently less healthy and emphasizes high-quality, minimally processed products with clear labeling and fewer additives. Despite facing market fluctuations and competition challenges, innovation and adaptability to shifting consumer needs remain crucial for the ongoing success of the plant-based protein sector^46^.

## Characteristics of plant-based meat ingredients

PBMAs harness biochemical similarities between plants and animals, with primary raw materials comprising proteins (20–50%), polysaccharides (2–30%), and fats (0–5%). These components are critical in determining the texture, while additional ingredients enhance flavor, color, and nutritional value. Effective PBMA formulation is paramount for obtaining desired texturization, binding, and dietary outcomes. In typical PBMA formulations, a blend of proteins, polysaccharides, and fats mimics the texture of meat^47^. Core ingredients include isolates and concentrates from soy, peas, and wheat. Recent developments have introduced alternative proteins such as potatoes, rice, lentils, and algae, improving texture, flavor, and nutrition^14^. Moreover, combinations like soy or pea proteins with cereal proteins (e.g., brown rice) improve the amino acid profiles^48^ and promote varied structural properties^49^, enhancing the overall quality of PBMAs.

### Proteins in PBMAs

Plant-based proteins significantly influence meat analogs’ structure, color, texture, and flavor, going beyond their nutritional role due to technical properties like solubility, emulsification, foam formation, and gelation^18^. Soy and pea proteins are prominent due to their cost-effectiveness, availability, and processing flexibility. They are often blended with wheat, potato, mung bean, and rice proteins to enhance nutrition and texture (Table 1). These plant-derived proteins, categorized into albumin (water-soluble), globulins (salt-soluble), prolamins (alcohol-soluble), and glutelins (soluble in weak acid/alkali solution), contribute to functional and nutritional qualities^17^.

Soy protein, a top choice in PBMAs, effectively mimics meat texture but may face issues like aftertaste and allergenicity. Derived from soybeans containing around 40% crude protein^50^, soy protein powder is processed through cleaning, grinding, dehulling, and de-oiling steps. Soy protein concentrate (SPC) and isolate (SPI) result from extracting soluble and insoluble carbohydrates from defatted soybean flakes, offering varying protein content levels^51^.

Pea protein is gaining prominence in the PBMA protein domain for its processing attributes^52^ and mild taste, rich protein quality, and amino acid profile^53^. However, it faces challenges in gel formation compared to soy protein. This disparity presents obstacles during the texturing phase of PBMA production, a crucial step in achieving the desired consistency and feel of the final product^54^. Innovative solutions like enzymatic modifications and polysaccharide conjugation are being explored to improve its techno-functional properties, such as gelling, emulsification, water retention, and oil retention^55^.

Wheat protein, known as gluten, is favored in PBMAs for its water retention capability, enhancing product juiciness and tenderness. Formulating PBMAs with wheat gluten involves blending it with other plant proteins, improving fibrous texture through protein cross-linking, and enhancing digestibility in extruded products^49^. This combination effectively leverages the nutritional synergy between grains and legumes, providing a comprehensive array of essential amino acids vital for human health^56^.

Potato protein, derived from potato juice, is recognized for its emulsifying properties and low allergenic risk, making it a versatile and appealing option for PBMA formulation, especially for those with dietary sensitivities^57^.

Rice protein, valued for its hypoallergenic traits and balanced amino acid profile, faces challenges in water solubility and gelling capability, which are being addressed through ongoing research to enhance its role as a texture modifier in PBMAs^58^.

Pulses are attracting significant attention as a burgeoning source of protein, offering a wealth of nutrients, including proteins, complex carbohydrates, vitamins, and minerals^59^. Lentil protein, praised for its gelling, emulsifying, and foaming properties, mirrors the functional traits of pea and soy proteins. Mung bean proteins excel in gel formation and water retention, albeit with variability in amino acid composition^60^. Faba beans stand out for their superior biological nitrogen fixation, promising enhanced agricultural productivity, reduced environmental footprint, and improved cost-efficiency, thus positioning them as a substantial addition to plant-based protein sources^61^.

Oilseed proteins, a by-product of edible oil processing, are emerging as eco-friendly options in the food industry due to their minimal anti-nutritional factors, abundance of essential amino acids, and high digestibility^62^. Notably, chia, hemp, and pumpkin seeds have been recognized for their health-promoting properties, including anti-inflammatory and cardioprotective effects, making them popular in health-focused food formulations^63^. An example of their applications is found in products like the Whole Burger from the plant-based meat brand Abbot’s, which incorporates chia seeds (https://vegoutmag.com/). This burger is reported to contain 22 g of protein per serving, potentially offering greater satiety and muscle health support compared to traditional veggie patties.

Pseudocereals like amaranth and quinoa are esteemed for their high-quality proteins loaded with essential amino acids and excellent gelling properties. These proteins significantly enhance the nutritional value and texture of PBMAs. Amaranth is renowned for its broad health benefits, while quinoa seeds are valued for their abundance of health-promoting flavonoids and antioxidants^64^. Isolated quinoa protein, in particular, is acclaimed for its superior emulsifying and gelling capabilities, broadening its application in food product development^65^.

Algae proteins, rich in essential amino acids and beneficial omega-3 fatty acids, are valued for their nutritional benefits in food products^66^. A New York-based alternative meat brand, Akua, utilizes sustainably farmed kelp to produce the Kelp Burger, an innovative addition to their product line which includes items like kelp jerky, known for its crispy exterior and warm interior (https://settingmind.com/). However, while seaweed-derived polysaccharides enhance texture, their unique sensory attributes can limit their application in specific food applications^67^.

Aquatic plants like duckweed (*Lemna minor*) are notable for their substantial protein content and rapid biomass production, closely mirroring the nutritional profile of animal proteins and often surpassing traditional plant proteins^68^. Duckweed is especially valued for its ribulose 1,5-bisphosphate carboxylase (RuBisCo) content, a crucial component known for its desirable properties and role as a precursor to bioactive peptides. However, the challenge lies in optimizing the extraction and isolation techniques for RuBisCo to fully leverage duckweed’s potential in food applications^69^. Meanwhile, products like Rubi Whisk™ have already harnessed similar plant-based advantages. Marketed as an egg replacer for PBMAs, Rubi Whisk™ offers enhanced fat binding, oil holding, and water-binding capabilities, providing a clean label, plant-based solution that enhances the structural integrity, moisture, and oil retention of allergen-free baked goods (https://www.plantiblefoods.com).

### Polysaccharides in PBMAs

Polysaccharides play a pivotal role in enhancing the functional and structural aspects of PBMAs. Derived from sources like potato, maize, wheat, cassava, pea, and rice, polysaccharides, including starches and flours, act as efficient fillers, improving texture and ensuring consistency across PBMAs. Fibers from peas, potatoes, and bamboo, along with polysaccharide gums such as xanthan gum and carrageenan, are instrumental in thickening PBMA products and reducing cooking loss. Their ability to retain water excellently and to form stable oil/water emulsions is crucial for attaining the desired consistency and mouthfeel in PBMAs^70^.

Incorporating dietary fiber into PBMAs enhances their nutritional value and imparts critical functional attributes. The capacity of dietary fiber to hold water is vital in mimicking the structure and texture of meat^71^. Including dietary fiber in PBMAs can lead to an increased fiber intake, potentially yielding health benefits like lowering LDL cholesterol and reducing risks associated with cardiovascular disease and obesity^72,73^.

Starch is a fundamental functional component in PBMAs, significantly impacting product yield, moisture retention, and texture modification^74^. Starch varieties with high amylopectin content, such as wheat and maize starch, are particularly effective in lending softness to PBMAs during the extrusion process, a vital characteristic for replicating the tenderness and bite of meat^75^. As such, starch is integral to PBMA formulations, serving as a primary structuring agent and significantly contributing to the texture and overall quality of the final product.

### Fats in PBMAs

Fats are essential in PBMAs for replicating traditional meats’ juiciness, tenderness, and flavor profiles. During heat processing, the onset of lipid oxidation plays a critical role in developing flavor compounds that enhance the meat-like taste^76^. In PBMAs, various plant-based oils, including coconut, sunflower, and avocado oils, are frequently used due to their health benefits compared to animal fats^70^. The desired characteristics of the final product guide the selection of these oils. Balancing unsaturated and saturated fatty acids is crucial in replicating the sensory qualities of meat. For example, semi-solid oils such as coconut oil can replicate the appearance of meat marbling. Still, their higher saturated fat content requires careful consideration to maintain the healthfulness of PBMAs^72^.

## Techniques for crafting PBMAs

The production of PBMAs involves a sophisticated process of protein extraction from sources like soy, pea, and wheat, structured into meat-like textures using techniques such as extrusion, shear cells, spinning, and 3D printing (Fig. 4a). Flavoring agents, fats, and additives are introduced to enhance the sensory appeal, with binding agents, texturizers, flavors, and coloring agents playing crucial roles in product enhancement.

### Structuring techniques

Plant-derived proteins are processed into forms that mimic the texture of traditional meat through various manufacturing techniques, notably LME and HME. LME transforms raw materials into a semi-solid blend, which is thermally treated in a cooking zone and then extruded through a long, slender die. This process produces TVP, characterized by an extended shelf life and a meat-like fibrous texture upon rehydration (Fig. 4b). LME typically results in greater extrudate expansion compared to HME. On the other hand, HME requires higher moisture content (40–70%) and an extended cooling die, creating layered, fibrous textures resembling whole-cut meat^40^. During HME, proteins are exposed to high temperatures, shear forces, and pressures, which facilitate the formation of cross-links and new protein-protein associations, ultimately yielding layered, fibrous textures that closely resemble the structure of muscle meat^77^. Additionally, optimal HME processing requires careful adjustment of various parameters, including the rotating speed of screw, barrel temperature, and feeding amount to achieve the desired textural outcomes. HME products, typically maintained in refrigerated or frozen states, closely resemble muscle meat in texture and appearance^78^.

Advanced structuring techniques such as 3D printing and shear cell technology enhance sensory experiences and nutritional value. Shear cell technology, still in its pilot phase, creates unique product structures by inducing flow within the product, resulting in distinctive fibrous textures. It encompasses phases similar to HME, including amalgamation and hydration, thermo-mechanical treatment, and cooling. While both HME and shear cell technology utilize shear forces to deform and align biopolymers, the resulting structures differ significantly in macroscopic appearance^42^. Extruded meat analogs exhibit a V-shaped pattern, where individual layers align parallel to the die wall. In contrast, the macrostructure of products obtained through shear cell technology displays distinct fibers oriented along the direction of the applied shear flow. Also, shear cell technology can potentially create PBMAs with greater thickness than those produced by HME methods^79^.

3D printing constructs products layer by layer, thereby enhancing sensory experiences and nutritional value^80^. This technology utilizes bio-inks derived from various animal and plant cells to fabricate cultivated meat (CM) that authentically mimics meat textures. It combines plant-based proteins and fibers using methods such as extrusion 3D printing, inkjet printing, and binder spraying, which are essential for developing plant protein-based edible inks from sources like soy protein isolate and wheat gluten^81,82^. For instance, an air-heating extrusion-based 3D printer uses a balanced blend of soy, wheat, and rice protein pastes to improve printing performance and create a meat-like layered structure^83^. Despite its potential, 3D printing faces challenges such as imprecision and suboptimal productivity, necessitating intensive research for successful commercialization^80^. Technical issues such as low viscosity, ink stability, nozzle clogging, and achieving adequate mechanical integrity are being addressed through technological improvements in extrusion techniques and material formulations. These enhancements aim to enhance the physicochemical properties of the final products^84^. Startups like Revo Foods and Nova Meat are utilizing extrusion 3D printing and integrating tissue engineering to hasten the development of both plant and cell-based meats^85^. Innovations in coaxial and dual extrusion are being developed to produce multi-material products that resemble whole-cut steaks, although challenges with ink flow characteristics remain^86^. Ongoing research efforts focus on optimizing formulations and nozzle sizes during 3D printing and cooking to enhance product attributes such as hardness, springiness, cohesiveness, and chewiness^87,88^. Additionally, blending technologies based on protein-protein interactions have been developed to improve fiber formation, thereby enhancing the rheological properties of bio-inks and boosting their performance in 3D printing applications^83^.

Additionally, the integration of scaffold materials such as edible microcarriers and fiber structures, which are often created using advanced manufacturing techniques, is crucial for tissue development and effective nutrient and oxygen transport within thicker structures^89^. To improve scaffold functionality, modifications like crosslinking with food-grade agents^90^ and the incorporation of bioactive polymers such as silk fibroin and gellan gum are used to enhance mechanical properties and cell adherence^91^. TVPs, derived from soy by-products, are used as scaffolding materials due to their structural versatility and nutritional benefits^20,92^, supporting efficient cell seeding and differentiation necessary for large-scale CM production.

Innovations in scaffold materials focus on reducing material costs and capital expenses while ensuring safety and optimizing bioprocessing systems for market readiness. As CM progresses towards commercial viability, integrating scaffold materials that minimize environmental impacts and align with consumer sustainability and ethical preferences is increasingly important^93^. Future directions will likely concentrate on enhancing scaffold interactions with cultured cells, reducing reliance on animal-derived materials, and aligning with sustainable food production goals. These initiatives aim to make CM more economically viable and environmentally sustainable, reflecting growing consumer demands for ethically produced alternatives.

Scaffold-free technologies like cell sheet engineering, utilize biocompatible materials (e.g., chitosan, alginate, gelatin) to enhance structural and mechanical properties and reduce costs by simplifying the cell expansion and harvesting process^94^. Cell sheet engineering allow cells to form monolayers and secrete their extracellular matrix, facilitating the noninvasive detachment and stacking of cell sheets to produce thick, high-density tissues without the need for animal-derived materials^95^. These technologies, combined with 3D printing, enable the integration of microcarriers into 3D printed hydrogels^96^ or directly within bioinks^97^. Combining cell sheet engineering with nutrient delivery platforms that incorporate algae-derived proteins and growth factors supports cell growth, reduces medium costs, and minimizes the use of animal-derived supplements^95^.

### Manufacturing & quality improvement technique

In PBMA production, the sophisticated amalgamation of plant proteins, various food additives, and state-of-the-art technologies is crucial for enhancing flavor and appearance, with ingredient synergy playing a pivotal role in overall quality enhancement and addressing product deficiencies. Techniques like extrusion transform these ingredients through denaturation and gelatinization, followed by reassembly into a structured network via bond modification such as noncovalent and disulfide bonds. This process typically promotes phase separation, forming molecular aggregates within a continuous protein matrix, essential for mimicking meat-like textures.

Binders are essential for manufacturing intricate meat analogs, enhancing texture, color, flavor, processing quality, and nutritional values. Ingredients such as soy or pea proteins alone may be inadequate for creating viscoelastic networks in emulsion-type meat analogs, necessitating the use of binders to foster cohesive and adhesive interactions, such as hydrogen bonding or electrostatic interactions^98^. These interactions help maintain component cohesion, fortify emulsion stability, curb oil leakage, and adhere to the TVP particles. Common binders like methylcellulose, known for its hydroxyl groups, solidify upon heating and revert to a viscous state upon cooling^99^. Other prevalent binders, including pectin, carrageenan, guar gum, cellulose, xanthan, and locust bean gum, contribute unique rheological properties that influence gelation, thickening, and texture^100^. Edible gum, notably carrageenan, and pectin, are often incorporated into soy protein meat substitutes, enhancing taste, texture, hardness, and chewiness, or electrostatically interacting with soy protein to bestow viscoelasticity and maintain stable physical properties under high-temperature shear^101^.

Achieving palatability is fundamental to PBMA’s market success, yet replicating the complex flavor profile of traditional meat poses significant challenges^100^. Key components such as iron, lactate, and inosine 5’-monophosphate are crucial for mimicking the authentic flavors of raw meat, while cooking-induced Maillard reactions and lipid oxidation further enrich the flavor^102^. PBMAs often require larger amounts of flavor enhancers such as savory yeast extract (YE), nucleic acids, and sugars compared to conventional meat. Spices and herbs are also used to emulate the rich flavors of processed meats and to mask any undesirable tastes from legume proteins^14^. Overcoming the beany flavor associated with plant proteins involves inactivating lipoxygenase and neutralizing flavors through fermentation or β-cyclodextrin^103^. Ingredients such as hydrolyzed vegetable protein (HVP) and YE are crucial in PBMA formulations^104^, with HVP producing a strong meat-like flavor when heated with sugars and yeast autolysis, and YE enhancing umami and kokumi flavors upon heating^105^.

The visual allure of meat analogs is paramount for customer acceptance. While traditional meat’s color is primarily attributed to myoglobin^106^, PBMAs often employ soy protein or gluten, resulting in hues that differ from meat’s expected red or brown. To mimic natural meat colors and marbling, innovations in plant-based pigments and ingredients like beetroot^107^ and leghemoglobin^108^ are used, although concerns about genetically modified origins are driving the development of safer, cost-effective alternatives to traditional coloring agents like titanium dioxide.

The manufacturing and quality improvement of PBMAs involves integrating various ingredients and technologies to enhance structural, flavor, and visual qualities. This includes optimizing key extruder operating parameters such as moisture content, barrel temperature, and screw speeds, which are crucial for proper texturization^87^. These parameters are part of a complex multi-input-output system^109^ that significantly impacts the texture and physicochemical properties of the final products^87,110^.

Meat substitution, both partial and complete, often use plant-based ingredients, with some commercial products incorporating animal proteins like dairy and eggs^52,92^. Brands like Hungry Jack’s® (v2foods™Australia), Impossible™ Burger (Impossible™ Foods, U.S.), the Beyond Burger® (Beyond meat®, U.S.), and Chicken free chicken® (Sunfed®, New Zealand) use a mix of protein sources (soy, pea, wheat) and carbohydrate (cellulose, methylcellulose, starch) to enhance water and lipid interactions, contributing to a meat-like texture^92^. However, the complexity of extrusion conditions—including high shear and temperature—can denature proteins, which complicates texture prediction and functionality. Additionally, factors like storage conditions, preprocessing, and harvesting variations significantly affect these properties, emphasizing the importance of understanding physiochemical interactions during extrusion^111^.

## Health consciousness in PBMA production

The rise of PBMA production has been supported by major conglomerates and fast-food chains, aiming to crater to a health-conscious market. However, maintaining a healthy image for PBMA poses challenges. To sustain their perception as a healthier alternative, a majority express willingness to increase PBMA consumption if their nutritional profile aligns with real meat. The perception of PBMA as highly processed food with potential health risks necessitates the development of new technologies and raw materials to address these concerns and shift consumer perceptions. A meticulous analysis of nutritional components and additives in PBMA can pave the way for healthier and safer alternatives.

### Nutritional quality in PBMAs

PBMAs strive to emulate the appearance and taste of meat, but nutritional quality can sometimes be compromised. Generally, PBMA products contain fewer calories, less total and saturated fat, and more dietary fiber than their meat counterparts. However, they may exceed sodium recommendations and lack essential micronutrients like iron, zinc, and vitamin B_12_^112^. Addressing these deficiencies through fortification can enhance the health profiles of PBMAs, positioning them as healthier alternatives

The surge in demand for plant-based proteins has spurred in-depth research into protein quality and bioavailability, assessed through the dietary indispensable amino acid score (DIAAS) and the protein digestibility-corrected amino acid score (PDCAAS)^113^. DIAAS measures the true ileal digestibility of essential amino acids in foods, while PDCAAS assesses protein quality based on digestibility and amino acid composition. To improve protein quality in PBMAs, integrating diverse plant sources and processing methods can enhance bioavailability by unfolding proteins, reducing antinutrients, and fostering optimal hydrolysis^114^. Although plant-based proteins often lack essential sulfur-containing amino acids^8,52^, HME cooking preserves lysine and enhances the bioavailability of other amino acids through thermal unfolding, making proteins more digestible^115^. Despite high moisture content potentially reducing the effectiveness of antinutritional factors (ANFs) deactivation^116^, the thermal and mechanical forces in HME cooking generally improve digestibility and reduce some ANFs, enhancing the nutritional profile of PBMAs^117^.

Antinutrients such as tannins, phenols, saponins, phytates, glucosinolates, and erucic acid can impede nutrient digestion and absorption^118^. Techniques like fermentation, soaking, and various processing methods (e.g., gamma irradiation, germination, heating, genomic technology, sonication, microwave, high-pressure processing, and electric field methodologies) can reduce their concentration and mitigate adverse effects^119^. For instance, extrusion processes enhance the bioavailability of essential amino acids while reducing ANFs, thereby improving the overall nutritional value of PBMAs.

While plant-based diets are typically low in sodium, PBMA products may have elevated sodium levels due to processing, posing health risks such as chronic kidney disease^120^ and increasing lithogenic risks among children and infants^121^. Reducing sodium content through natural seasonings and exploring sodium-curbing flavors and processing techniques can offer healthier PBMA options^122^.

Iron, zinc, and vitamin B_12_ are challenging to obtain in a meat-free diet, and their bioavailability in meat substitutes can be limited by factors like phytate content^123^. Addressing these micronutrient deficiencies is crucial, especially among specific demographic groups^124^. Vitamin B_12_, absent in plants, is an additional challenge, causing deficiencies, especially among vegetarians, vegans, pregnant women, or females in their reproductive years^125^. Fortifying PBMA products and advocating for nutrient-rich plant foods in diets can mitigate these challenges^126^. There is a critical need for education and guidelines centered on plant-based nutrition and fortification to ensure healthy and sustainable diets.

### The effect of PBMAs on human gut microbiome

The shift towards reduced meat consumption has increased interest in PBMAs, particularly among flexitarians concerned about meat’s association with cardiometabolic diseases and gut dysbiosis. Despite this interest, some consumers hesitate to adopt PBMAs, perceiving them as low-quality and highly processed, potentially harmful to gut microbiota. This underscores the necessity for targeted research into the complex relationship between meat intake and gut microbiota composition, which is crucial for human health^127^.

Recent studies demonstrate that PBMA consumption may beneficially impact the gut microbiome by increasing butyrate production and microbial diversity^128^. These effects are often attributed to the increased dietary fiber in PBMAs rather than their intrinsic properties. However, the limited scope of these studies, often with small sample sizes, necessitates further research with robust methodologies to substantiate these health implications comprehensively. For instance, research using the TIM gastrointestinal model showed that plant-based burgers (PB) influenced the lipemic response and gut microbiome differentially than beef^129^. PBs generally contain higher carbohydrate levels due to the addition of binders like potato starch, methylcellulose, and maltodextrin, which contribute to a meat-like texture, while typically having lower protein and fat content. Short-term PB intake significantly alters the gut bacterial colonization, increasing the Firmicutes to Bacteroidetes (F/B) ratio and affecting short-chain fatty acid (SCFA) profiles, indicating that the nutritional content and structure of food influence gut microbiota composition. However, it is unclear whether these phenotypes are ascribed to the types of meat or the abundance and species of components in PBMAs.

Observational and experimental studies have provided inconsistent results regarding the specific changes in gut microbiota composition at different taxonomic levels due to meat consumption^130^. These inconsistencies highlight the complexity of understanding how meat attributes, such as processing and cooking methods, impact gut microbiota^131^. This complexity underscores the need for well-designed randomized controlled trials and systematic reviews that control dietary variables more precisely, aiming to clarify the impacts of meat and PBMAs on gut microbiota and broader health outcomes^127^.

### Clean label emphasis in PBMA

PBMA formulations often include proteins, water, flavors, oils or fats, binders, and colorants. However, the presence of numerous additives to compensate for the functional limitations of eco-friendly proteins can lead to extensive ingredient lists, potentially deterring consumers who prioritize natural and transparent ingredients. The trend towards “clean label” products, emphasizing minimal and recognizable ingredients, is growing^132^. Such products resonate with consumers seeking transparency and naturalness in their food choices. Factors influencing consumer perceptions of “naturalness” include the origin of the food, processing methods, E-number labeling, and the presence or absence of chemicals or artificial additives^133^. While chemical additives like methylcellulose (E461) are commonly used in PBMAs as binders for their thickening and emulsifying properties, the demand for clean-label products has spurred the development of chemical-free alternatives such as pea protein and sugar beet pectin^134^. Hydrocolloids from natural polysaccharides like konjac glucomannan, κ-carrageenan, konjac mannan, and xanthan gum are increasingly used for their gelling, thickening, emulsifying, and stabilizing properties, contributing to the desired textural properties of PBMAs^133^. Regulatory changes, such as the European Commission’s ban on certain additives, have prompted the food industry to seek safe, natural whitening ingredients for PBMA formulations. Companies are simplifying ingredient lists and using HME and proprietary processes with multi-functional proteins to enhance the texture and sensory qualities of PBMAs, aligning with the clean label trend and catering to consumer preferences for natural and transparent products. Beyond Meat’s IV platform exemplifies the evolution of PBMA into the third generation. It replaces traditional fats with avocado oil to reduce saturated fat and increase the smoke point, making it suitable for BBQ. Additionally, it cuts sodium by 20% and boosts nutritional value with new ingredients like red lentils and fava bean protein. This innovation has earned it the distinction of being the first plant-based meat to receive clean label project certification.

## Challenges and opportunities in PBMA development

Taste is crucial in PBMA purchasing decisions, and enhancing the quality of plant-based proteins can reduce off-notes and improve flavor. However, replicating the texture of animal-based meat poses significant challenges, particularly as some PBMAs lack essential amino acids and micronutrients. Addressing ANFs is vital to enhance the nutritional profile of these products. PBMA production costs currently surpass those of traditional meats, affecting affordability and consumer adoption. Recent advances include the Feature-Augmented Principal Component Analysis to model the extrusion process precisely^135^. This approach has produced six mathematical models and the first visualization software for HME, enhancing equipment setup, parameter adjustments, and quality control.

**Fig. 5:**
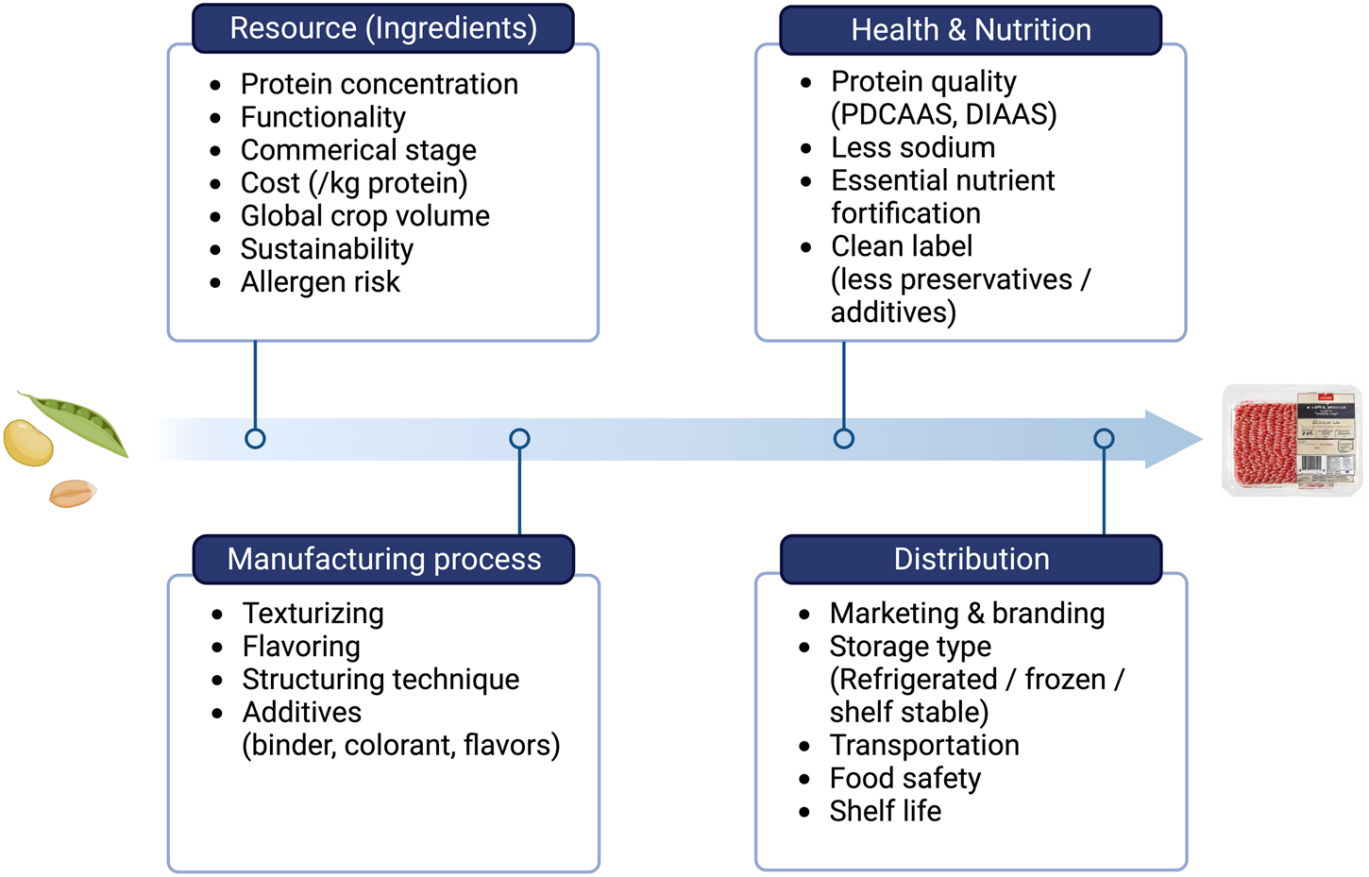
Roadmap highlighting the developmental stages of novel PBMAs. This infographic presents a step-by-step guide outlining the critical criteria for each phase of PBMA manufacturing and distribution.

Moreover, PBMAs provide a sustainable alternative to traditional meat, requiring fewer natural resources and producing less environmental waste. As production scales up and technologies evolve, costs are expected to decrease, making PBMAs more accessible and reducing their environmental impact. Despite growing popularity, continuous improvement in taste, texture, and nutritional value is necessary (Fig. 5). Effective marketing and educational campaigns can enhance consumer awareness and acceptance. By tackling challenges like undesirable flavors and nutrient deficiencies, the industry can improve consumer perceptions and contribute significantly to a sustainable food ecosystem.

## Data Availability

All data supporting the findings of this study on global meat production and consumption trends are openly available at https://ourworldindata.org.
